# An Alternative Method to Facilitate cDNA Cloning for Expression Studies in Mammalian Cells by Introducing Positive Blue White Selection in *Vaccinia* Topoisomerase I-Mediated Recombination

**DOI:** 10.1371/journal.pone.0139349

**Published:** 2015-09-30

**Authors:** Hiroshi Udo

**Affiliations:** Department of Biology, Graduate School of Science, Kyushu University, Fukuoka, Japan; George Mason University, UNITED STATES

## Abstract

One of the most basic techniques in biomedical research is cDNA cloning for expression studies in mammalian cells. *Vaccinia* topoisomerase I-mediated cloning (TOPO cloning by Invitrogen) allows fast and efficient recombination of PCR-amplified DNAs. Among TOPO vectors, a pcDNA3.1 directional cloning vector is particularly convenient, since it can be used for expression analysis immediately after cloning. However, I found that the cloning efficiency was reduced when RT-PCR products were used as inserts (about one-quarter). Since TOPO vectors accept any PCR products, contaminating fragments in the insert DNA create negative clones. Therefore, I designed a new mammalian expression vector enabling positive blue white selection in *Vaccinia* topoisomerase I–mediated cloning. The method utilized a short nontoxic LacZα peptide as a linker for GFP fusion. When cDNAs were properly inserted into the vector, minimal expression of the fusion proteins in *E*. *coli* (harboring *lacZΔM15*) resulted in formation of blue colonies on X-gal plates. This method improved both cloning efficiency (75%) and directional cloning (99%) by distinguishing some of the negative clones having non-cording sequences, since these inserts often disturbed translation of *lacZ*α. Recombinant plasmids were directly applied to expression studies using GFP as a reporter. Utilization of the P2A peptide allowed for separate expression of GFP. In addition, the preparation of *Vaccinia* topoisomerase I-linked vectors was streamlined, which consisted of successive enzymatic reactions with a single precipitation step, completing in 3 hr. The arrangement of unique restriction sites enabled further modification of vector components for specific applications. This system provides an alternative method for cDNA cloning and expression in mammalian cells.

## Introduction

*Vaccinia* topoisomerase I-mediated cloning, known as TOPO cloning by Invitrogen, has been widely used in biological and medical research. It was originally developed by Shuman [1], which revolutionized the way to clone PCR-amplified DNAs. It is quick and efficient without relying on restriction enzymes and DNA ligases. Since the cloning procedure is very simple, this method is suitable for large scale cloning as well [2].

The cloning method is based on unique properties of *Vaccinia* topoisomerase I [3]. The enzyme recognizes a specific pentapyrimidine sequence of 5’-(C/T)CCTT↓-3’ and hydrolyzes the phosphodiester bond at the 3’-end of its recognition site [4]. Upon cleavage, a covalent bond is formed between DNA and the enzyme (3’-phosphate of the DNA and tyrosine 274 of the enzyme), and the nicked strand is relaxed in the mean time. The phosphor-tyrosyl bond energy is then utilized to relegate to the 5’-hydroxyl end of the cleaved strand, and the enzyme is released from the DNA after reaction. When the enzyme reacts with a linear double-stranded DNA having its recognition site near the 3’-end, a short terminal fragment is released from the nicked strand, and the enzyme remains covalently bound to the DNA. This enzyme-DNA complex is stable, and is able to recombine with an exogenous DNA having a 5’-hydroxyl end.

In the original cloning procedure, insert DNAs were first reacted with *Vaccinia* topoisomerase I, and the resulting enzyme-insert DNA complex was then added to the dephosphorylated vector DNA [1]. This procedure was later modified to a well-known format, in which the enzyme was linked with the vector DNA (TOPO vector) to accept insert DNAs having 5’-hydroxyl ends. This is particularly suitable for cloning of PCR-amplified DNAs, since chemically synthesized primers have a 5’-hydroxyl end. If TOPO vectors are ready, recombination can be done by simply adding PCR products.

A variety of TOPO vectors are commercially available today. However, the most popular applications may be cDNA cloning for expression studies in mammalian cells. Since expression vectors require cDNAs to be inserted in the correct orientation, directional TOPO cloning vectors are suitable (such as a pcDNA3.1 directional TOPO cloning vector from Invitrogen). Oriented insertion was facilitated by the inclusion of four bases “CACC” (corresponding to a portion of Kozak sequence [5]) at the 5’-end of the insert DNA and also at the cloning site of the vector. Furthermore, pcDNA3.1 allows expression of cloned cDNAs in mammalian cells under control of a CMV promoter [6]. This is time and cost-efficient since conventional strategies involve two steps, cDNA cloning into a cloning vector and subcloning into an expression vector.

I attempted to clone mouse cDNAs using the pcDNA3.1 directional TOPO cloning vector. Although a number of cDNAs were successfully obtained, I found that the cloning efficiency was generally lower. RT-PCR does not always yield a single major band of interest and the product often includes nonspecifically amplified fragments. As a result, excised gel slices are more or less contaminated. Since TOPO vectors accept any PCR products as inserts, contaminating fragments create negative clones, thereby reducing the efficiency. Moreover, I found that some cDNAs were inserted in the opposite orientation, and the percentage of directional cloning was about 80%.

The aim of this study is to create a simple, efficient, and convenient cloning vector that can be used for expression analysis in mammalian cells. It was designed to meet the following requirements: 1) it’s a mammalian expression vector, 2) it enables simple and efficient cDNA cloning, 3) it also expresses a reporter in mammalian cells (such as GFP in a fused or separate form), and 4) it can be modified for different application. In attempts to improve cloning efficiency, I have introduced positive blue white screening into *Vaccinia* topoisomerase I-mediated recombination. By distinguishing some of the negative clones arising from insertion of non-cording sequences or reverse insertion of cDNAs, this method improved the overall efficiency as well as directional cloning. The obtained plasmids were directly used for expression analysis using GFP as a reporter. In addition, preparation of the *Vaccinia* topoisomerase I-linked vectors was considerably simplified. This system is user-friendly and I hope it contributes to biomedical research involving cDNA cloning and expression in mammalian cells.

## Materials and Methods

### Cloning vectors

For construction of pCMVlac-dirTopo-AU1/P2A-αGFP (4.85 and 4.89 kbp in length, the vector DNA sequences in S1 File), pEGFP-C2 (Clontech) was used as a vector backbone, in which the regions between *Nhe*I and *Xba*I sites (815 bp, including EGFP and cloning sites) and between *Ssp*I and *Eag*I sites (447 bp, the promoter region of a kanamycin resistance gene) were modified. A PCR-amplified fragment (~1.0 kbp) was inserted at *Nhe*I-*Xba*I sites of pEGFP-C2, which contained a modified *E*. *coli* lactose promoter/operator (*lacPO*), cloning site, AU1 or P2A sequence, modified *lacZα*, and *GFP*. The cloning site (65 bp, flanked by *Acc*III and *Bam*HI sites) and AU1/P2A sequences (18 bp for AU1 [7, 8] or 57 bp for P2A [9, 10], flanked by *Bam*HI and *Sal*I sites) were created by combination of oligonucleotides. The modified *lacPO* (57 bp, flanked by *Nhe*I and *Bsp*EI sites) was prepared by PCR using pUC19 as a template, in which two point mutations were introduced to eliminate ATGs and to reduce its promoter activity. The modified *lacZα* (132 bp, corresponding to the region from Ile 4 to Arg 47 of *E*.*coli* β-galactosidase, flanked by *Sal*I and *Eco*RI sites) was PCR-amplified from the *E*.*coli lacZ* gene, in which silent mutations were introduced to remove several restriction sites and to adjust codon usages for mammals. *GFP* (714 bp, flanked by *Eco*RI and *Xho*I/*Xba*I sites) was PCR-amplified from pEGFP-C2. The upstream region of a kanamycin resistance gene (the 447-bp region between *Ssp*I and *Eag*I sites in pEGFP-C2) was rearranged by placing the bacterial promoter just upstream of the open reading frame. For cloning mouse cDNAs, a vector having a different fluorescent marker (mKO, monomeric Kusabira Orange, Medical & Biological Laboratories) was used to meet the needs of subsequent experiments. To construct ampicillin resistant vectors (pUC-dirTopo-AU1/P2A-αGFP, 3.75 and 3.79 kbp in length, the vector DNA sequences in S1 File), the 1.9-kbp *Ssp*I-*Afl*III fragment from pCMVlac-dirTopo-AU1/P2A-αGFP (the region spanning from the CMV promoter to SV40 polyadenylation signal) was inserted at *Aat*II (followed by blunting)-*Afl*III sites of pUC19. Vectors without *lacPO* were constructed by deleting the region between *Nhe*I and *Bsp*EI sites in pCMVlac-dirTopo-AU1-αGFP and pUC-dirTopo-AU1-αGFP. Constructs were verified by DNA sequencing. Plasmid DNAs were purified with PureLink HiPure plasmid miniprep or midiprep kit (Invitrogen). Concentration of purified DNA was about 1 μg/μl (OD_260_/OD_280_ = 1.8–2.0). The plasmids DNAs were deposited in Addgene (https://www.addgene.org). The accession numbers are #61971 for pCMVlac-dirTopo-AU1-αGFP, #61972 for pCMVlac-dirTopo-P2A-αGFP, #61973 for pUC-dirTopo-AU1-αGFP, and #61974 for pUC-dirTopo-P2A-αGFP

### Purification of *Vaccinia* topoisomerase I

A bacterial expression vector of *Vaccinia* topoisomerase I, pET-Top1B [11], was generously provided by Dr. Shuman. His-tagged *Vaccinia* topoisomerase I was expressed via T7 expression system in *E*. *coli* [12] and purified as reported previously [11] with minor modification. The recombinant protein was bound to TALON metal affinity resin (Clontech), washed with 5 mM imidazole in the extraction buffer (50 mM sodium phosphate [pH 7.0], 0.3 M NaCl, 0.1% Triton X-100), and eluted with 150 mM imidazole in the extraction buffer.

### Preparation of *Vaccinia* topoisomerase I-linked vector DNAs

A detailed protocol is provided in S1 File. The purified vector DNA (5 μg) was first digested with 10 unit of Nt.*Bsp*QI (a nicking enzyme [13], New England Biolabs) in a 50-μl reaction volume for 1 hr at 50°C. The enzyme was heat denatured at 80°C for 20 min. The vector was then digested with 15 unit of *Eco*RV for 1 hr at 37°C. For the 10-μl reaction (5-kbp vector DNA, 1 μg or 0.3 pmole), 0.4 μl of 0.5 M EDTA, and 1 μl of purified *Vaccinia* topoisomerase I (32 kDa enzyme, 0.2 μg or 6 pmole) were added and reacted for 5 min at 37°C. On ice, the vector DNA (11 μl) was mixed with 9 μl H_2_O, 20 μl of 30 mM MgCl_2_, and 20 μl of 30% polyethylene glycol (PEG, average molecular weight of 8,000, Sigma), and centrifuged at 14,000 rpm for 20 min at 4°C. The supernatant was completely removed and enzyme-linked vector DNA was promptly recovered by dissolving in 10 μl of 1x Topo buffer with EDTA (50 mM Tris-HCl [pH 7.5], 100 mM NaCl, 2.5 mM EDTA). The vector concentration was 20–40 ng/μl as judged by agarose gel electrophoresis. *Vaccinia* topoisomerase I-linked vector DNAs were used for cloning or stored at -20°C.

### Preparation of cDNAs

Reverse transcription-polymerase chain reaction (RT-PCR) was performed to obtain mouse cDNAs (growth factors and transcription factors, size: 0.24–4.67 kbp, average size: 1.25 kbp). The cDNAs were synthesized from the total RNA (derived from embryonic mouse brains) *in vitro* with a PrimeScript II 1st strand cDNA synthesis kit (Takara), and were diluted 5-fold with sterile water. The PCR reaction mixture (25 μl) consisted of 17 μl of H_2_O, 5 μl of 5x HF buffer (New England Biolabs), 0.5 μl of 10 mM dNTPs, 0.5 μl of cDNA, 1 μl of specific primers (a mixture of forward and reverse primers, 25 pmol/μl each), 0.75 μl of DMSO, and 0.25 μl of Phusion High-Fidelity DNA polymerase (2 U/μl, New England Biolabs). Primer sequences were designed according to the Invitrogen’s protocol. Synthetic oligonucleotides were purchased from Integrated DNA Technologies. Forward primers were 25 nucleotides in length including 4 extra nucleotides of “CACC” at the 5’-end and 21 nucleotides corresponding to the 5’-region of the open reading frame (starting from the initiation codon). Reverse primers were 21 nucleotides in length, corresponding to the 3’-region of the open reading frame without the termination codon. The reaction cycle, time, and temperature for PCR were as follows: one cycle of 1 min at 98°C, 35 cycles of 10 sec at 98°C, 20 sec at 57~60°C, and 30~90 sec at 72°C, and one cycle of 5 min at 72°C and holding at 4°C. Annealing temperature and extension time were varied depending on cDNAs. After PCR, five μl of 6x DNA loading dye was added to the PCR products and the samples were resolved by agarose gel electrophoresis in Tris-acetate-EDTA (TAE) buffer (0.7, 1, or 2% agarose gel containing ethidium bromide at 1 μg/ml gel, run at 100 V for 30 min). Gel pieces containing the PCR product of the expected size were excised (a sample image in S1 Fig). DNA fragments were purified with QIAEXII gel extraction kit (Qiagen) and finally eluted in 20 μl of H_2_O. The recovery of DNA fragments was confirmed by agarose gel electrophoresis. The concentration of purified DNAs was 10–20 ng/μl. For test cloning of mKO, the DNA fragment was PCR-amplified from phmKO2-MC1 (Medical & Biological Laboratories) with specific primers (forward primer 5’-CACCATGGTGAGCGTGATCAAG-3’ and reverse primer 5’-GGAGTGGGCCACGGCGTC-3’). PCR and fragment purification of mKO were performed in the same manner (a sample image in S1 Fig).

### *Vaccinia* topoisomerase I-mediated cloning

A detailed protocol is provided in S2 File. *Vaccinia* topoisomerase I-mediated cloning was performed in a 2.5-μl reaction volume consisting of 0.5 μl of the enzyme-linked vector (10~20 ng), 0.5 μl of the purified PCR-amplified cDNA (5~10 ng), 0.5 μl of 5x Topo buffer (250 mM Tris [pH 7.5], 500 mM NaCl, 12.5 mM MgCl_2_), and 1 μl of H_2_O. The reaction mixture was incubated for 10 min at room temperature. On ice, it was then added to 50-μl of *E*.*coli* DH5α competent cells and incubated for 30 min (other *E*. *coli* strains harboring *lacZΔM15* may be used, competent cells were prepared by Hanahan’s methods [14], the competency at 1 x 10^6^ colony forming unit/μg DNA/100 μl cells). Cells were heat-shocked at 42°C for 1 min and then chilled on ice for 2 min. Five hundred μl of 2xYT medium was added and incubated for 2–3 hr (for kanamycin-resistant plasmids) or 1 hr (for ampicillin-resistant plasmid) at 37°C. One-half of bacterial suspension (250 μl) was spread on a X-gal plate (LB agar [15] supplemented with 40 μg/ml X-gal and 30 μg/ml kanamycin or 100 μg/ml ampicillin, no addition of isopropyl-β-D-thiogalactopyranoside). Bacterial plates were incubated for 18–22 hr at 37°C to obtain blue colonies. Prolonged incubation (over 22 hr) of bacterial plates should be avoided, because the white colonies become bluish as a result of nonsense suppression in *E*.*coli*. In many cases, light-blue colonies did not contain inserts. In this protocol, a moderate number of transformants (~90 colonies/plate) were obtained. When the vectors harboring an ampicillin-resistance gene were used (pUC-dirTopo-AU1/P2A-αGFP), the number of transformants was significantly increased by 4~8-fold. Rearrangement of the upstream region of a kanamycin resistance gene (in an attempt to improve its expression in *E*.*coli*) did not increase the number of transformants.

### Confirmation of recombinant DNAs

Blue colonies on X-gal plate were picked, dipped in the PCR solution, and transferred to new plates (LB plates supplemented with 30 μg/ml kanamycin or 100 μg/ml ampicillin). The reaction mixture for colony PCR (25 μl/sample) was composed of 20 μl of H_2_O, 2.5 μl of 10x ThermoPol buffer (New England Biolabs), 0.5 μl of 10 mM dNTPs, 1 μl of specific primers (a mixture of forward and reverse primers, 25 pmol/μl each, the same primers used for RT-PCR), 0.75 μl of DMSO, and 0.2 μl of *Taq* DNA polymerase (5 U/μl, New England Biolabs). The PCR program consisted of one cycle of 2 min at 94°C, 35 cycles of 20 sec at 94°C, 20 sec at 57°C, and 60~180 sec at 72°C (extension time were adjusted), and one cycle of 5 min at 72°C and holding at 4°C. Five μl of 6x DNA loading dye was added to the reaction mixture and the amplified DNAs were analyzed by agarose gel electrophoresis in TAE buffer at 100 V for 30 min (0.7, 1, or 2% agarose gel containing 1 μg/ml ethidium bromide). If there was no band at the expected size, other colonies on the original plates were examined in a similar fashion. For the clones having the insert DNA of the expected size, bacteria on master plates were cultured in 5 ml of 2xYT medium supplemented with 30 μg/ml kanamycin or 100 μg/ml ampicillin overnight at 37°C. Plasmid DNAs were purified with PureLink HiPure plasmid miniprep kit (Invitrogen) and eluted in 50 μl of TE buffer. Concentration of purified DNA was about 1 μg/μl. The insert DNAs were confirmed by DNA sequencing. Samples were prepared with a BigDye terminator v3.1 cycle sequencing kit (Applied Biosystems), and examined by a sequencer 3130 Genetic Analyzer (Applied Biosystems).

### Cell culture and transfection

HEK293 cells [16] were grown in the culture medium consisting of Dulbecco’s modified Eagle medium (DMEM, Gibco), 10% fetal bovine serum (Sigma, heat-inactivated at 56°C for 30 min), and antibiotics (Gibco, 100 unit/ml penicillin and 100 μg/ml streptomycin) in a humidified 5% CO_2_ incubator at 37°C. Cells were maintained in a ϕ90 mm culture dish containing 8 ml of the culture medium. For imaging, ϕ15 mm glass coverslips (Matsunami, acid- and alkaline-washed) coated with rat tail collagen (Roche, 0.5 mg/ml in 0.2% acetic acid, 50 μl/coverslip for 1 hr at room temperature) were placed in a 12-well culture dish (one coverslip per well) containing 1 ml/well of the culture medium, and HEK293 cells was added to each well at 1x10^4^ cells/cm^2^. On the following day, the cells were transfected with pCMVlac-Pax6-AU1-αGFP, pCMVlac-Pax6-P2A-αGFP, pCMVlac-dirTopo-AU1-αGFP, pCMVlac-dirTopo-P2A-αGFP, or pEGFP-C2, in which two μg of DNAs in 50 μl DMEM and 1 μl of lipofectamine 2000 (Invitrogen) in 50 μl DMEM were mixed, left for 15 min, and then added to each well. The culture medium was exchanged at 24 hr. Forty-eight hr after transfection, cells were briefly fixed with 4% paraformaldehyde in phosphate-buffered saline (PBS) for 5 min, washed with PBS, and treated with 0.1% Triton X-100 in PBS for 5 min. Samples were then incubated with blocking solution (3% donkey serum in PBS) for 1 hr, and reacted with an anti-Pax6 antibody (1:200, mouse monoclonal, Santa Cruz) overnight at 4°C. Cells were washed 6 times with PBS for 5 min each, and reacted with a Cy5-conjugated anti-mouse IgG goat antibody (1:200, Jackson Immunoresearch) and propidium iodide (PI, 10 μg/ml, Sigma) in blocking solution for 2 hr at room temperature. After washing 6 times with PBS for 5 min each, coverslips were mounted onto slide glasses. Fluorescent images were acquired with a confocal microscope LSM510 (Zeiss). Argon and helium lasers at 488, 543, and 633 nm were used for excitation of GFP, PI, and Cy5, respectively. A 100x oil (N.A. 1.5) objective lens was used for examination. Imaging conditions were optimized to avoid saturation and photobleaching, and all images were acquired under the same setting. Images were analyzed with LSM Image Browser (Zeiss).

### Western blotting

HEK293 cells in ϕ35 mm culture dishes at 60% confluence were transfected with DNA-lipofectamine complex (a mixture of 4 μg of pCMVlac-dirTopo-AU1-αGFP, pCMVlac-dirTopo-P2A-αGFP, pCMVlac-Pax6-AU1-αGFP, or pCMVlac-Pax6-P2A-αGFP and 2 μl of lipofectamine 2000 in 200 μl of DMEM per dish) and the medium was refreshed at 24 hr. Forty-eight hr after transfection, cells were rinsed with PBS, chilled on ice, and lysed with 100 μl/dish of 0.1% Triton X-100. One-half of the lysed cells were centrifuged at 10,000 g for 10 min at 4°C to further separate into nucleus and cytosol fractions. Protein samples (whole cell, nucleus, and cytosol fractions in SDS-sample buffer, ~1 μg/μl, 10 μl/lane) were resolved by SDS-PAGE and blotted onto PVDF membranes. A pre-stained marker (P7708S, New England Biolab) was used to determine apparent molecular weights of proteins. The membranes were soaked in blocking buffer (5% skim milk in T-PBS [0.05% Tween-20 in PBS]) for 1 hr and incubated with an anti-β-tubulin antibody (1:5000, mouse monoclonal, Sigma) or anti-Pax6 antibody (1:1000, mouse monoclonal, Santa Cruz) in blocking solution at 4°C overnight. The membranes were washed 3 times with T-PBS for 10 min each, and reacted with a horseradish peroxidase (HRP)-conjugated anti-mouse IgG goat antibody (1:10000, Jackson Immunoresearch) in blocking buffer. The membranes were washed 3 times with T-PBS for 10 min each, and then washed twice with distilled water. Finally, the membranes were soaked in 1 ml/membrane of Lumi-Light chemiluminescence reagent (Roche) for 1 min, wrapped with a plastic bag, and analyzed by an image scanner LAS-3000 (Fuji Film).

### Statistical analysis

All data are expressed as mean ± standard error of the mean. Statistical analyses were performed with unpaired Student’s *t*-test or one-way ANOVA (multiple comparisons by Tukey-Kramer’s post-hoc test, if applicable). In some cases, Mann-Whitney *U*-test was used as a non-parametric analysis. A value of *P* < 0.05 was considered to be significant.

## Results

### Designing of the vector

**Fig 1:**
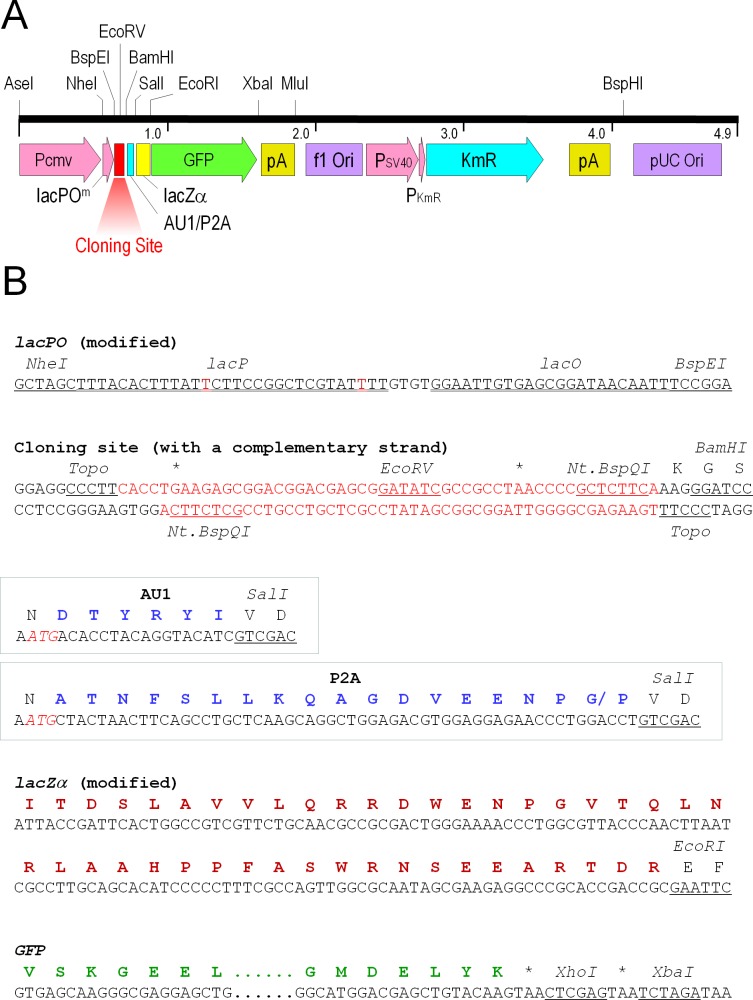
Cloning vectors. **A**. A linear map of pCMVlac-dirTopo-AU1/P2A-αGFP (4.85 and 4.89 kbp in length). A PCR-amplified cDNA is inserted at the cloning site via *Vaccinia* topoisomerase I-mediated recombination. A cytomegalovirus promoter (P_CMV_) and a modified *E*.*coli* lactose promoter/operator (lacPO^m^) are located upstream of the cloning site, which drive gene expression in mammalian cells as well as in *E*.*coli*. The cloning site is followed by the AU1 epitope or the P2A sequence (AU1/P2A), *lacZα* (encoding a peptide of *E*. *coli* β-galactosidase Ile4-Arg47, used as a linker) and *GFP* (encoding a green fluorescence protein, used as an expression marker). A SV40 polyadenylation signal (pA) is located downstream of *GFP*. The plasmid carries a kanamycin/neomycin resistance gene (KmR) under a SV40 early promoter and a KmR promoter, followed by a herpes simplex virus thymidine kinase polyadenylation signal (pA). The vector contains replication origins of f1 and pUC. Some of the unique restriction sites are shown. The numbers indicate the position of nucleotides in kbp. **B**. The vector sequence between *Nhe*I and *Xba*I sites. The modified *lacPO* (between *Nhe*I and *Bsp*EI sites) contains two point mutations (in red letters) to eliminate ATGs and also to reduce the promoter activity. The cloning site (between *Bsp*EI and *Bam*HI sites, its complementary sequence is also shown) contains two Nt.*Bsp*QI recognition sites, two *Vaccinia* topoisomerase I (Topo) recognition sites, and a unique *Eco*RV recognition site. Upon cloning, the fragment between two Topo recognition sites (in red letters) is replaced with a PCR-amplified insert DNA. The AU1 epitope or P2A sequence (between *Bam*HI and *Sal*I sites) is located right after the cloning site (in blue boxes). An out-of-frame ATG is created just before the sequences (in red italics). Capital letters above the nucleotide sequence represent single-letter amino acid codes (above the 2^nd^ position of each codon). A slash in the P2A peptide sequence indicates the self-cleaving site. The modified *lacZα* (between *Sal*I and *Eco*RI sites) contains silent mutations to eliminate several restriction sites and to adjust codon usages. *GFP* (between *Eco*RI and *Xho*I sites, encoding EGFP from Clontech) lacks its initiation codon. Asterisks indicate termination codons.

To create an efficient and convenient cloning vector suitable for expression studies in mammalian cells, I designed a plasmid, pCMVlac-dirTopo-AU1-αGFP (Fig 1). In attempts to enhance cloning efficiency, positive blue white selection was incorporated into *Vaccinia* topoisomerase I–mediated cloning. A modified *E*.*coli lac* promoter/operator (*lacPO*, 57 bp in length) was placed just downstream of a CMV enhancer/promoter (*Pcmv*, 590 bp in length). Two point mutations were introduced to *lacPO* to reduce its promoter activity for limited expression in *E*.*coli*, and to eliminate ATGs so as not to attenuate eukaryotic translation. Downstream of the cloning site, an AU1 epitope, *lacZα*, and *GFP* were inserted in-frame. LacZα served as a linker between the cloned cDNA and *GFP*. To block translation of *lacZα-GFP* under the modified *lacPO*, in-frame ATGs were removed and two in-frame stop codons (within the cloning site) and an out-of-frame ATG (just before the AU1 sequence) were placed. Therefore, only when a cDNA fragment is properly inserted into the vector, the cloned gene is expressed as a GFP fusion protein with a LacZα linker peptide. Its expression in *E*.*coli* strains harboring *lacZΔM15* (e.g. DH5α) will lead to formation of blue colonies on X-gal plates via α-complementation of β-galactosidase [17].

### Test cloning of mKO for comparative analyses

**Fig 2:**
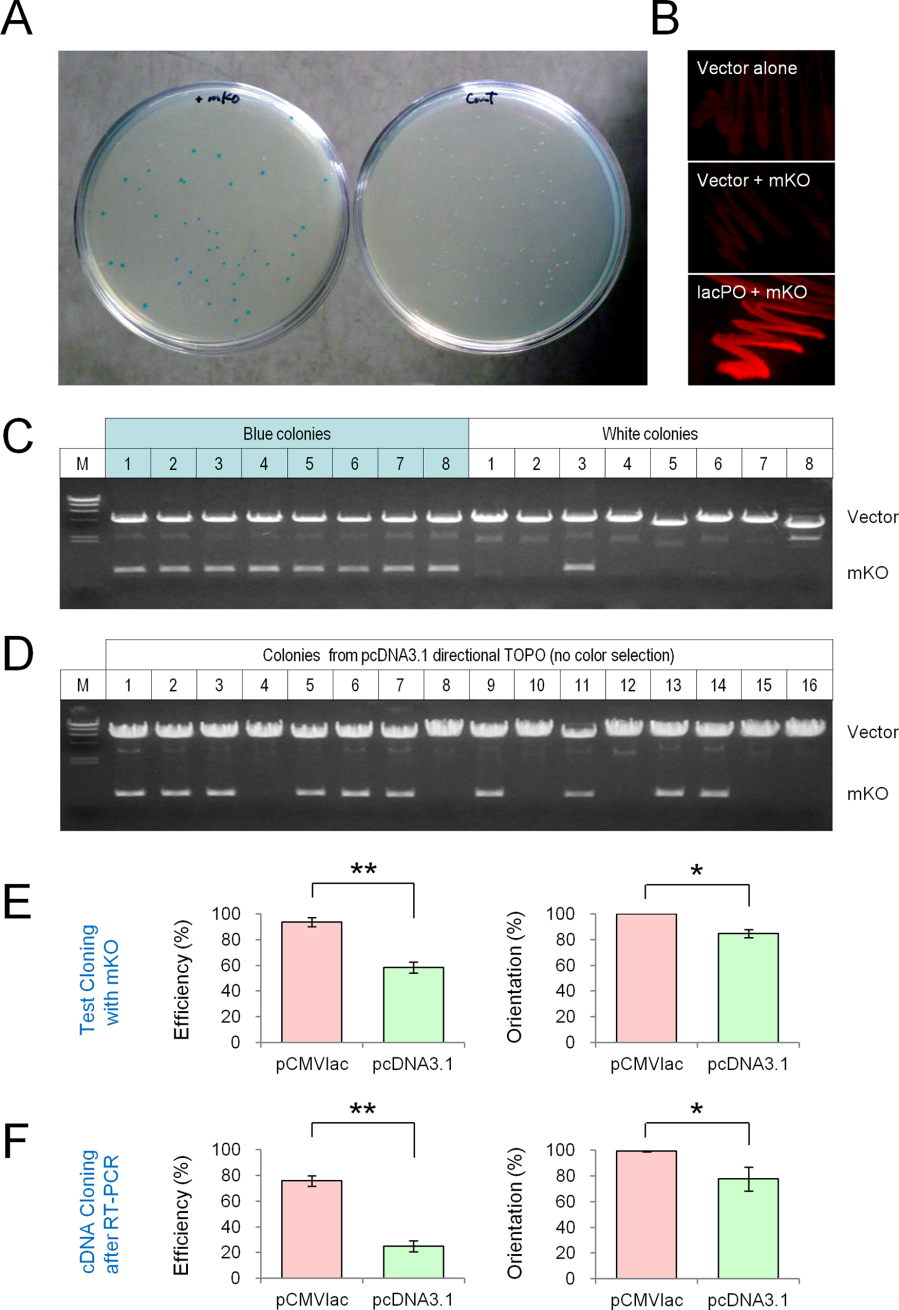
Positive blue white screening. **A.** Test cloning of monomeric Kusabira Orange (mKO) in pCMVlac-dirTopo-AU1-αGFP. The image shows transformants on X-gal plates (left: vector with mKO, right: vector alone). When mKO was cloned, the gene product was expressed as a GFP fusion protein with a LacZα linker peptide, resulting in formation of blue colonies on X-gal plate via α-complementation of β-galactosidase. In this test, the ratio of blue colony was 80%. **B.** Limited gene expression in *E*. *coli*. Images represent mKO fluorescence from bacterial streak culture after 2 days of incubation (top: vector alone, middle: vector with mKO, bottom: mKO under a wild-type *lacPO*). Due to green auto-fluorescence of bacteria, orange fluorescence was used for comparison. The mKO fluorescence under the wild-type *lacPO* was clearly observed, but it was barely visible under the modified *lacPO* in the cloning vector. Blue white selection was more sensitive than fluorescence detection. **C.** Analysis of blue and white colonies in mKO test cloning. The plasmid DNAs were isolated from eight blue and white colonies, and digested with *Bsp*EI and *Bam*HI (top and bottom bands represent the vector and insert fragments, respectively). Sequencing analysis indicated that all blue colonies contained mKO in the correct orientation. Most of the white colonies had no insert (self-ligation) but some contained mKO in the opposite orientation (white colony #3 in this test). **D.** Test cloning of mKO using a pcDNA3.1 directional TOPO cloning vector. Plasmid DNAs were isolated from 16 colonies and digested with *BamHI* and *Xho*I. In this test, ten clones contained mKO, but two of them had mKO in the opposite orientation. **E.** Cloning efficiency and insert orientation in test cloning with mKO. The results of pCMVlac-dirTopo-AU1-αGFP (pCMVlac) and a pcDNA3.1 directional TOPO vector (pcDNA3.1) were shown. * *P* < 0.05, Mann-Whitney *U*-test. ** *P* < 0.01, Student’s *t*-test. **F.** Cloning efficiency and insert orientation in cDNA cloning after RT-PCR. The newly designed vector performed better than the pcDNA3.1 directional TOPO vector. * *P* < 0.05, ** *P* < 0.01, Mann-Whitney *U*-test.

To test the feasibility of this method, monomeric Kusabira Orange (mKO, an orange fluorescent protein, 654 bp in length) was cloned into pCMVlac-dirTopo-AU1-αGFP via *Vaccinia* topoisomerase I-mediated recombination (Fig 2A). After transformation of *E*.*coli* DH5a, blue and white colonies were formed on X-gal plates, while the vector control produced only white colonies. The percentage of blue colonies was 76.4 ± 2.8% (n = 12). I examined thirty-two blue colonies and found that all clones were correct, bearing in-frame directional insertion of mKO. The analysis of white colonies showed that most of them had no insert (self-ligated products) but some contained mKO in the wrong orientation (Fig 2C). It was somewhat surprising that there were no blue colonies having mKO in the opposite orientation (total 48 blue colonies were tested), although reverse insertion of mKO should create as many as ten “ATG”s (two out-of-frame ATGs and eight in-frame ATGs for *lacZα*). However, it also introduces twenty stop codons, contributing to termination of translation. This implies that insertion of non-coding sequences into the vector may perturb proper translation of *lacZα*, thereby producing white colonies on X-gal plates.

In this experiment, I found that it is not practical to use GFP fluorescence for identifying positive colonies. Although the gene product was expressed as a GFP fusion protein in bacteria, green fluorescence from blue and white colonies were indistinguishable even after 24 hr of incubation (the signals were mostly attributed to auto-fluorescence from bacteria, not shown). For comparison, mKO fluorescence from bacterial streak culture was analyzed in Fig 2B. The orange fluorescence of cloned mKO was very weak, which was not significantly different from that of vector alone (adjusted mean fluorescent intensity, mKO: 1.7 ± 1.0 vs. vector alone: 1.4 ± 1.0, n = 4; non-significant, Tukey-Kramer post-hoc test). As a positive control, mKO fluorescence with a wild type *lacPO* was clearly visible, which was 15-fold brighter than that with a modified *lacPO* (lacPO-mKO: 25.8 ± 6.0, n = 4; *F*(2,9) = 15.42, *P* < 0.01, one-way ANOVA; lacPO-mKO vs. mKO, *P* < 0.01, Tukey-Kramer post hoc test). This result suggests that bacterial expression of gene products is very limited, and that blue white selection is more sensitive than detection of GFP fluorescence.

Test cloning of mKO was performed with a pcDNA3.1 directional TOPO cloning vector as well (Fig 2D). About 70% of transformants contained mKO, but 15% of them had inserts in the opposite orientation, reducing the efficiency to about 60%. In Fig 2E, the cloning efficiency and insert orientations were analyzed. The pCMVlac-dirTopo-AU1-αGFP vector performed better than the pcDNA3.1 directional TOPO vector in the overall efficiency (pCMVlac: 93.8 ± 3.6%, pcDNA3.1: 58.3 ± 4.2%, n = 3 tests for each vector; *t*(4) = 6.43, *P* < 0.01, Student’s *t*-test) and directional cloning (pCMVlac: 100 ± 0.0%, pcDNA3.1: 84.7 ± 3.2%, n = 3 tests for each vector; *U* = 0, *Z* = -1.96, *P* < 0.05, Mann-Whitney *U*-test).

### Improved efficiency in cDNA cloning

The cloning system was applied to mouse cDNAs (growth factors and transcription factors). RT-PCR was performed and the gel-purified fragments were used as inserts (S1 Fig). In this attempt, 106 out of 126 genes (84%, cDNAs that produced blue colonies on X-gal plates) were successfully cloned. The percentage of blue colonies (excluding light blue colonies) on X-gal plates was 29.8 ± 2.3% (it was 76% in test cloning). The cloning efficiency was slightly reduced to 75.1 ± 3.5%, but the percentage of directional cloning remained high (99.5 ± 0.5%). When a single blue colony was analyzed, 78.1% of them were correct. Therefore, analysis of just one or two colonies was sufficient in many cases.

I initially used the pcDNA3.1 directional TOPO vector for cDNA cloning. Although 15 out of 18 genes (83%) were successfully obtained, it involved analysis of many colonies due to the increased number of negative clones (containing no inserts or smaller fragments). The efficiency was reduced to 25.1 ± 4.3% in cDNA cloning (58% in test cloning), which was significantly lower than 75% with the new vector (*U* = 440.5, *Z* = -5.09, *P* < 0.01, Mann-Whitney *U*-test). In contrast, the percentage of directional cloning was 77.8 ± 9.2%, similar to the levels observed in test cloning (84%), but pCMVlac outperformed pcDNA3.1 in this aspect as well (*U* = 711, *Z* = -2.18, *P* < 0.05, Mann-Whitney *U*-test).Therefore, incorporation of blue white selection into *Vaccinia* topoisomerase I–mediated recombination was found to be effective in cDNA cloning,

### Expression of *Pax6* in mammalian cells with or without P2A

**Fig 3:**
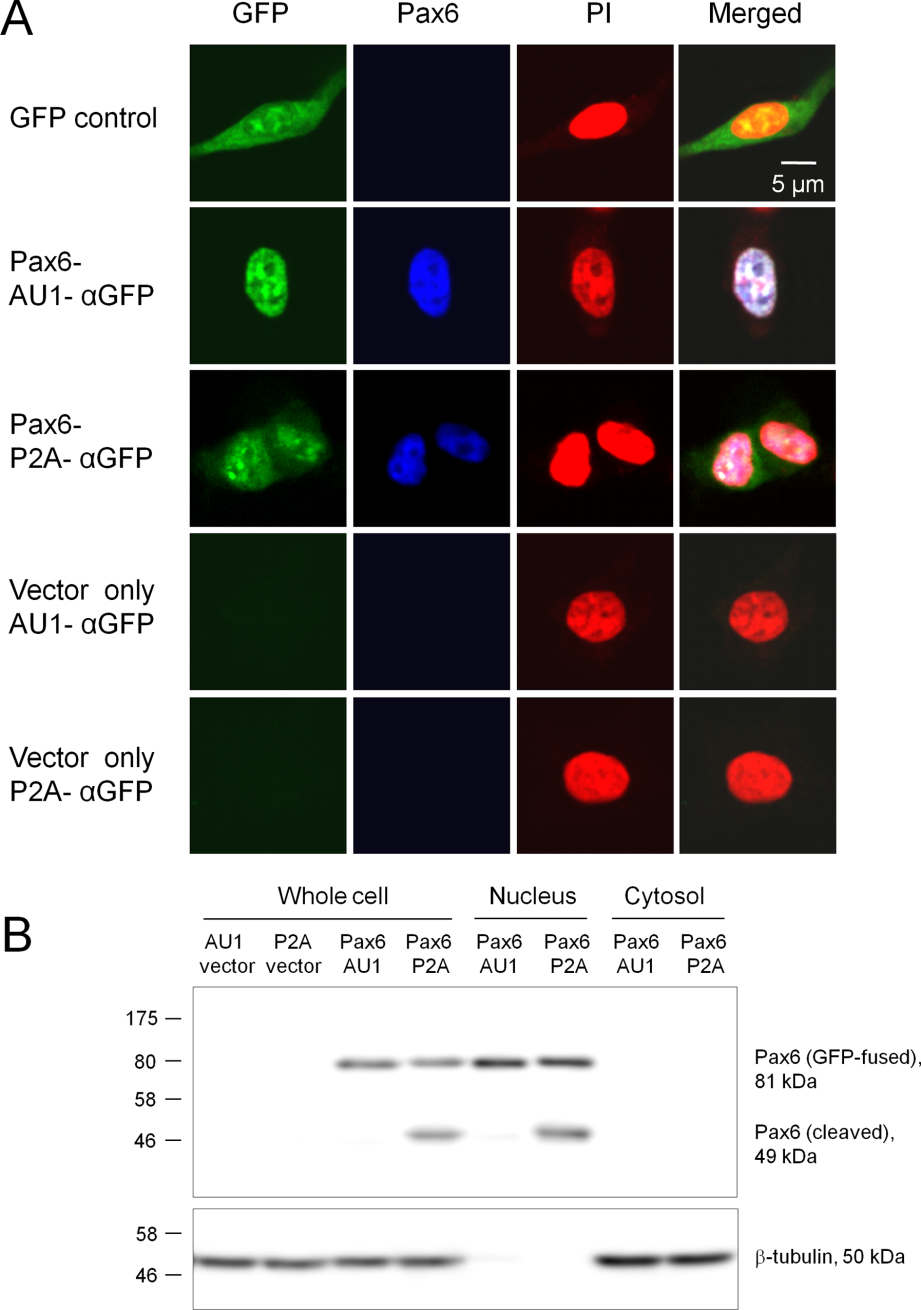
cDNA expression in mammalian cells. **A.** Fluorescence imaging of mammalian cells expressing Pax6. Mouse *Pax6* (a transcription factor involved in neurogenesis) was cloned in pCMVlac-dirTopo-AU1-αGFP and pCMVlac-dirTopo-P2A-αGFP, and expressed in HEK293 cells. Vector controls and pEGFP-C2 (GFP control) were also included in this analysis. Expression and subcellular localization of the gene products were identified by GFP fluorescence (green). Cell nuclei were visualized with propidium iodide (PI, red), and Pax6 was immunostained (blue). Pax6 was exclusively localized in the nuclei. When Pax6 was expressed using the P2A vector, GFP fluorescence tended to diffusely distribute in the cytoplasm as a result of self-cleavage at P2A. Scale bar, 5 μm. **B.** Western blotting of transfected cells. Pax6 was expressed using the AU1 and P2A vectors in HEK293 cells. Whole cell, nucleus, and cytosol fractions were prepared and probed with an anti-Pax6 antibody (top panel) or anti-β-tubulin antibody (bottom, as an internal control). The vector controls did not have any Pax6 staining. GFP-fused Pax6 was detected at 81 kDa and localized exclusively in the nucleus. About one-half (53%) of Pax6-P2A-αGFP, but not Pax6-AU1-αGFP, was self-cleaved, which produced a 49 kDa band (cleaved Pax6) localizing in the nucleus.β-tubulin (50 kDa) was absent from the nucleus fraction. The numbers on the left indicate the size of pre-stained marker proteins in kDa.

Recombinant plasmids can be directly used for expression studies in mammalian cells. Expression of the gene product can be easily monitored by GFP fluorescence. In this study, I also designed a plasmid containing a P2A sequence [9, 10] in place of AU1 (see Fig 1). With this vector, fusion proteins may be self-cleaved at the P2A site to release the fused LacZα-GFP. Fig 3A shows the expression of mouse *Pax6* (a transcription factor involved in neuronal differentiation) in HEK293 cells. When *Pax6* was expressed using the AU1 vector, both GFP fluorescence and Pax6 immunostaining were detected in the cell nuclei (indicated by PI staining). In contrast, when *Pax6* was expressed using the P2A vector, GFP fluorescence tended to diffusely distribute throughout the cytoplasm, while Pax6 immunostaining was observed only in the nucleus. Expression of GFP alone visualized the whole cell without any Pax6 signals. Vector controls only showed PI staining in the nucleus under 30–40% transfection efficiency. The efficiency of self-cleavage by P2A was examined by Western blotting (Fig 3B). The result indicated that 55.2 ± 3.6% (n = 3 tests) of Pax6-P2A-αGFP (81 kDa) was self-cleaved, producing a 49 kDa band of Pax6. Both GFP-fused and cleaved Pax6 were detected in the nucleus fraction (β-tubulin, as an internal control, was absent in the nucleus fraction). It suggests that at least a part of LacZα-GFP was cleaved off by P2A. Although green fluorescence of GFP was generally weaker with the P2A vector, it still functioned as an expression marker for transfected cells. The P2A vector may be useful when the functionality of the fusion protein is concerned.

I also confirmed that expression of *lacZα-GFP* per se does not cause cytotoxicity in HEK293 cells (percentages of PI-positive cells, mock control: 0.74 ± 0.26%; AU1-αGFP: 1.51 ± 0.32%; P2A-αGFP: 1.02 ± 0.31%; n = 8 areas, 52–223 cells/area, *F*(2,21) = 1.71, *P* = 0.21 by one-way ANOVA). Both *lacZ* and *GFP* have been widely utilized as reporters in mammalian systems for many years (such as recombinant embryonic stem cells and genetically modified mice). Therefore, utilization of *lacZα*, a small fragment of *lacZ*, and *GFP* in the newly designed vectors will not affect cell phenotypes.

### Simplified preparation of *Vaccinia* topoisomerase I-linked vectors

**Fig 4:**
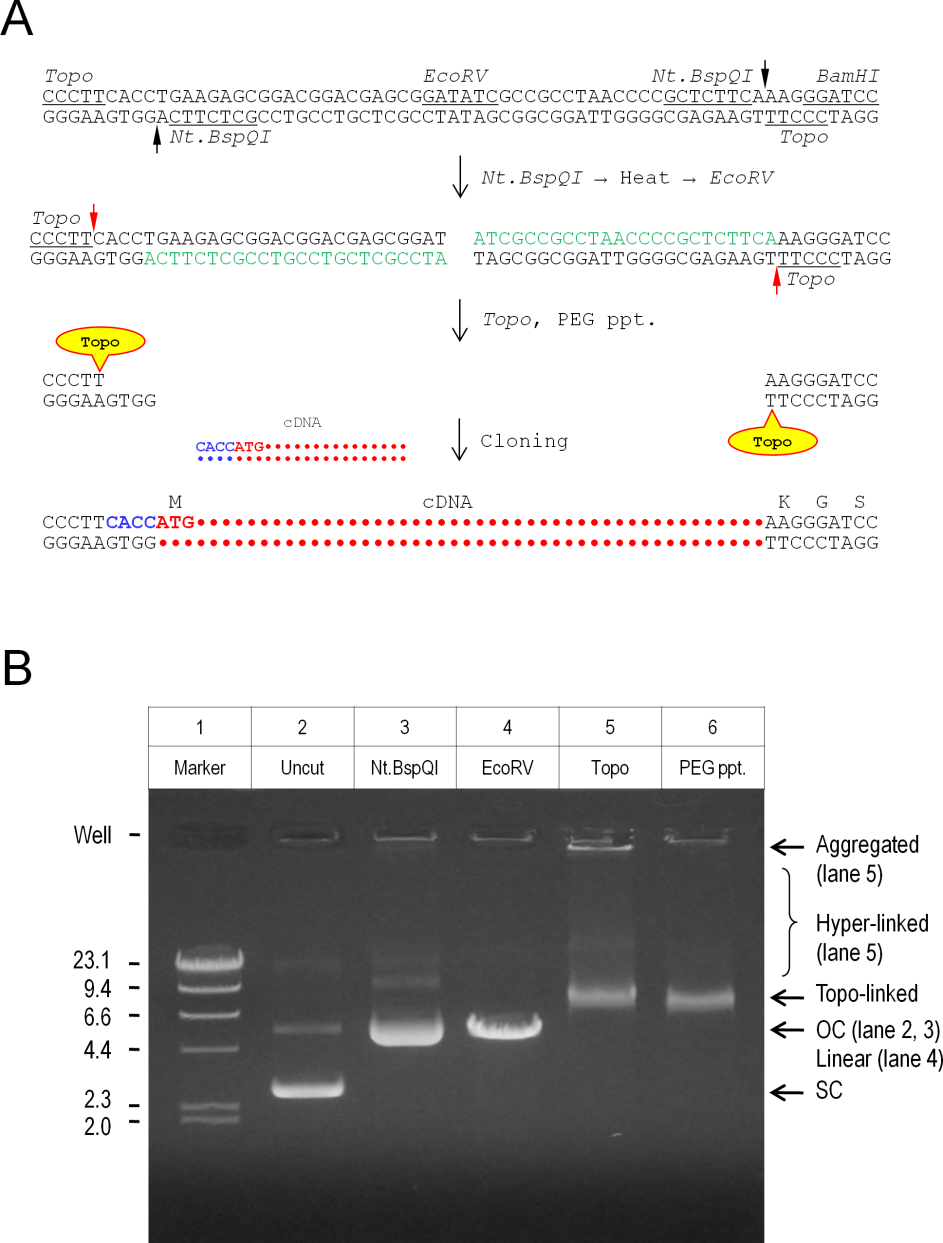
Simplified preparation of *Vaccinia* topoisomerase I-linked vector. **A.** Schematic diagram of vector preparation. The procedure consisted of successive enzymatic reactions with a single precipitation step. The vector DNA was first digested with Nt.*Bsp*QI (5’-GCTCTTCN↓-3’, black arrows indicate the nicking sites) for 1 hr at 50°C, heated at 80°C for 20 min, and linearized with *Eco*RV (5’-GAT↓ATC-3’) for 1 hr at 37°C. These treatments produced two 24-nucleotide fragments (in green letters) which remained bound to the vector DNA (Tm 75.5°C). Purified *Vaccinia* topoisomerase I (5’-CCCTT↓-3’, red arrows indicate the nicking sites) was then added and linked with the vector DNA for 5 min at 37°C. Finally, the enzyme-linked vector was precipitated with 10% polyethyleneglycol (PEG, Mw 8,000) at 14,000 rpm for 20 min at 4°C. The whole process was completed in 3 hr. **B.** Agarose gel electrophoresis of the vector DNA at each step of preparation. The uncut vector was comprised of super coiled (SC) and open circular (OC) conformations (lane 2). Digestion with Nt.*Bsp*QI yielded nicked plasmids, which migrated at the open circular position (lane 3). *Eco*RV digestion linearized the vector DNA, producing a single band at 4.85 kb (lane 4). Addition of *Vaccinia* topoisomerase I resulted in band shift of the vector DNA (lane 5). Some DNAs were hyper-linked (smeared appearance) or aggregated in the loading well. Precipitation with 10% PEG recovered the enzyme-linked vector, but most of the hyper-linked and aggregated DNAs were removed (lane 6). Marker, λ/*Hin*dIII (the numbers indicate the size of DNA fragments in kbp).

In the course of experiments, preparation of *Vaccinia* topoisomerase I-linked vector was streamlined. It consisted of successive enzymatic reactions with a single precipitation step, and the whole process was completed in 3 hr (Fig 4A). For preparation, the vector DNA was first digested with Nt.*Bsp*QI, linearized with *Eco*RV, and then linked with *Vaccinia* topoisomerase I followed by precipitation with 10% PEG. Fig 4B illustrates agarose gel electrophoresis of the vector DNA at each step of preparation. The uncut vector consisted of super-coiled and open circular forms. After digestion with Nt.*Bsp*QI, the nicked plasmids migrated at the open circular position. *Eco*RV digestion linearized the vector DNA, producing a single band on the gel. Addition of *Vaccinia* topoisomerase I resulted in gel-shift, suggesting that the enzyme was bound to the DNA. Some DNAs were hyper-linked (smeared appearance), or aggregated in the loading well. The enzyme-linked vector was recovered by PEG precipitation, removing most of the hyper-linked and aggregated DNAs. The recovery was 20–40% of the starting material. The present preparation is simpler than the method described by Heyman et al. [2], which required not only restriction digestions but also ligation of oligonucleotides, multiple phenol/chloroform extractions, and gel-purification of the vector DNA.

## Discussion

In this study, I attempted to construct an efficient and convenient vector suitable for cDNA cloning and expression studies in mammalian cells. Positive blue white selection was introduced into *Vaccinia* topoisomerase I-mediated cloning, which significantly improved the overall efficiency and directional cloning. The obtained plasmids were applied to expression analysis without the need for subcloning of cDNAs. Subcellular localization of the gene product was readily monitored by GFP fluorescence, and utilization of P2A allowed for separate expression of GFP.

TOPO cloning is an excellent method to clone PCR amplified DNAs. However, the vectors accept any PCR products as inserts and contaminating fragments reduce the cloning efficiency. In fact, this is a common problem of a variety of cloning methods that utilize PCR products as inserts. In particular, RT-PCR does not always produce the DNA fragment of interest as the major product and it often generates non-specific fragments (a sample image in S1 Fig). In many cases, gel purification cannot completely remove such byproducts. This explains why the pcDNA3.1 directional TOPO vector performed well in test cloning (58%) but was less efficient in cDNA cloning (25%). The advantage of the new system is the ability to distinguish some of the negative clones via color selection. Insertion of a non-cording sequence at the cloning site tends to disturb translation of *lacZα*, which in turn gives rise to white colonies on X-gal plates. Improvement in directional cloning was achieved by the same mechanism (99%, vs. about 80% in the pcDNA3.1 directional TOPO vector). Interestingly, a preference for insert direction was unchanged in test cloning and cDNA cloning (for both pCMVlac and pcDNA3.1), suggesting that reduced efficiency in cDNA cloning was attributed to contaminating fragments rather than reverse insertion of cDNAs. Although incorporation of positive selection facilitated cDNA cloning, negative clones were not completely excluded (94% in test cloning vs. 73% in cDNA cloning), showing the limitation of this technique. An additional benefit of this method is the capability to circumvent the background problem. I found that long-term storage of TOPO vectors results in a reduction of cloning efficiency due to the increased ratio of negative clones harboring self-ligated vectors. With the new vector, such negative clones will form white colonies on X-gal plates and may not directly affect the cloning efficiency (although the blue/white ratio will be reduced).

The selection method per se is an independent technique, and, therefore, it does not alter the specific features of TOPO cloning such as insert size-dependent cloning efficiency (not shown, see also [2]). In this study, I introduced positive blue white selection into TOPO cloning, but the vectors illustrated here are also applicable to Clontech’s In-Fusion cloning [18] (preliminary experiments). The efficiency of directional cloning by In-Fusion was very high by virtue of longer PCR primers (each requiring a 15-bp overlap with the vector sequence). However, similar to TOPO cloning, In-Fusion cloning also accepts any PCR products as inserts, and the utilization of positive blue white selection may improve the cloning efficiency.

Blue white screening was developed about 40 years ago [17], and has been widely utilized in molecular cloning. This method is based on the functional inactivation of β-galactosidase by cloning external DNAs into the structural gene (a type of negative selection). In 1996, Keese and Graf introduced positive screening based on α-complementation of β-galactosidase [19]. They devised a cloning vector carrying *lacZα* under *lacPO* without its Shine-Dalgarno sequence and initiation codon (a 14-nucleotide deletion resulting in translational inactivation of *lacZα*). Therefore, incorporation of the deleted 14-nucleotide sequence into the reverse primer and subsequent cloning of the PCR-amplified DNA at the 5’-end of *lacZα* led to a gain of function. This ingenious method took advantage of polycistronic expression in bacteria. However, inclusion of at least 14 nucleotides into PCR primers is somewhat inconvenient and nonspecifically amplified PCR products may create pseudo-positive blue colonies on X-gal plates. In this study, I simply utilized LacZα as a linker for GFP fusion, since it is sufficiently small and nontoxic to cells. This system was proven to be functional and provided an additional advantage that transformants having frame-shift or nonsense mutations as well as transformants having non-cording sequences are more likely excluded.

However, positive blue white selection is rarely used. This may be due to “leaky” bacterial expression, in which the vector alone or self-ligated vector tends to form pseudo-positive blue colonies on X-gal plates. One possibility is nonsense suppression in bacteria [20]. I found that removal of the initiation codon for *lacZα*or addition of multiple in-frame stop codons upstream of *lacZα* cannot prevent its translation. Since there are many nonsense suppressors (for amber, ocher, and opal) in *E*.*coli*, it is practically difficult to overcome such leakiness by mutagenesis. Therefore, I introduced an “out-of-frame” initiation codon, which turned out to be effective in attenuating the translation of *lacZα* (however, prolonged incubation of bacterial plates still turned white colonies to light blue). Leaky bacterial expression may also arise from promoter-like sequences. Bacterial promoters are relatively short (conserved sequences in -35 and -10 boxes [21]), and similar sequences may be found in stretches of DNA. For example, CMV enhancer/promoter (about 590 bp in length) also contains two promoter-like sequences (5’-TTGACGTCAATGGGTGGAGTATTTACGGTAAA-3’, 5’-TTGACGCAAATGGGCGGTAGGCGTGTACG-3’, underlines indicate -35 and -10 boxes respectively, Softberry software). Intriguingly, I found that the vector lacking *lacPO* also worked, although blue color development was delayed by 4 hr. Moreover, polycistronic readthrough is a common feature in bacteria (e.g. operon transcription). In short, nonsense suppression and promoter-like sequences as well as polycistronic readthrough make it difficult to completely block leaky expression in bacteria, which may have been an obstacle in development and utilization of positive blue white screening.

The newly designed vectors improved the cloning efficiency. However, there are two potential limitations in this technique. 1) Prokaryotic expression: This method relies on bacterial expression of cloned cDNAs, although the expression level was very limited (GFP fluorescence from blue colonies is hardly visible). Therefore, if the gene product is toxic to bacteria, such clone may be hard to obtain. However, as described above, leaky bacterial expression is difficult to eliminate, and cloning of toxic genes with conventional vectors may encounter similar problems. 2) Enzyme availability: For vector preparation, two molecules of *Vaccinia* topoisomerase I should bind to one molecule of the vector DNA (considering only two ends at the cloning site, note that there are as many as 27 enzyme recognition sites in the vector sequence). Therefore, a considerable amount of enzyme is required for vector preparation in comparison with standard enzymatic reactions (e.g. plasmid relaxation assay). In this study, one μg of the vector DNA (5 kbp in length, 0.3 pmole) was reacted with 0.2 μg of *Vaccinia* topoisomerase I (32 kDa, 6 pmole, stoichiometrically 20 times more than the vector DNA). I previously tested a commercial enzyme (Epicentre, supplied at 10 unit/μl) for vector preparation, but it was not successful. It was likely attributed to insufficient amounts of the enzyme used. It is anticipated that sufficient quantities of the enzyme will become commercially available in the near future. However, it is important to note that positive blue white screening is not necessarily associated with TOPO cloning, and it can be used in combination with other systems such as In-fusion cloning.

The vectors shown here can be modified for specific application. Unique restriction sites are placed at boundaries of each functional unit. For example, *Pcmv* can be replaced with a different promoter using *Ase*I and *Nhe*I. If necessary, a desirable epitope may be inserted between *Bam*HI and *Sal*I sites. *GFP* can be exchanged with a different reporter using *Eco*RI and *Xho*I. The vectors may be converted to TA cloning [22] by simply adjusting the position of Nt.*Bsp*QI sites. Although I designed these vectors for mammalian expression, the principle of positive blue white selection can be applied to different systems. This technique is simple and user-friendly. I hope that it facilitates biomedical research involving cDNA cloning for expression studies in mammalian cells.

## Supporting Information

S1 Fig Electrophoresis of DNA fragments. Sample images of PCR products for mKO test cloning and RT-PCR products for cDNA cloning (0.7% agarose gel electrophoresis).
(PDF)

S1 File Vector sequences. DNA sequences for pCMVlac-dirTopo-AU1/P2A-αGFP and pUC-dirTopo-AU1/P2A-αGFP
(PDF)

S2 File Protocols. Protocols for cDNA cloning and vector preparation
(PDF)
