# Supplementary material for: An Alternative Method to Facilitate cDNA Cloning for Expression Studies in Mammalian Cells by Introducing Positive Blue White Selection in Vaccinia Topoisomerase I-Mediated Recombination
Source: PLoS One. 2015 Sep 30;10(9):e0139349. doi: 10.1371/journal.pone.0139349 (PMC4589362; doi:10.1371/journal.pone.0139349)

S1 Fig. Sample images of PCR or RT-PCR products

A. PCR products for mKO test cloning

DNA	Marker	mKO
(bp)	λHindIII	657
Product		Yes
Amount		+++
Size		OK

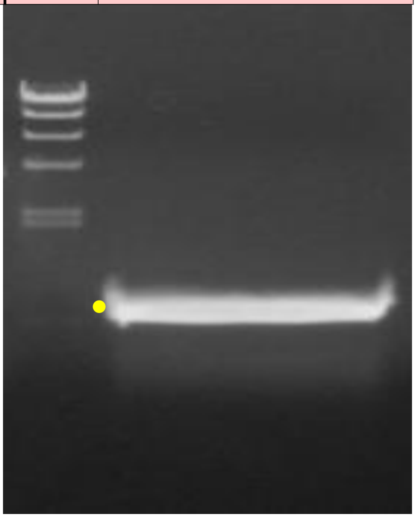

B. RT-PCR products for cDNA cloning

DNA	BDNF	Marker	Cux1	Gli1	Glis3	Gsc	Gsx1	Hes5	Hoxa1	Hoxb1	Irx5	Klf16
(bp)	774	λHindIII	4281	3336	2808	771	786	504	1011	894	1455	756
Product	Yes		No	Yes	Yes	No	Yes	Yes	Yes	Yes	Yes	No
Amount	+++		-	±	+++	-	+	+	++	±	+++	-
Size	OK		-	OK	OK	-	OK	OK	OK	OK	OK	-

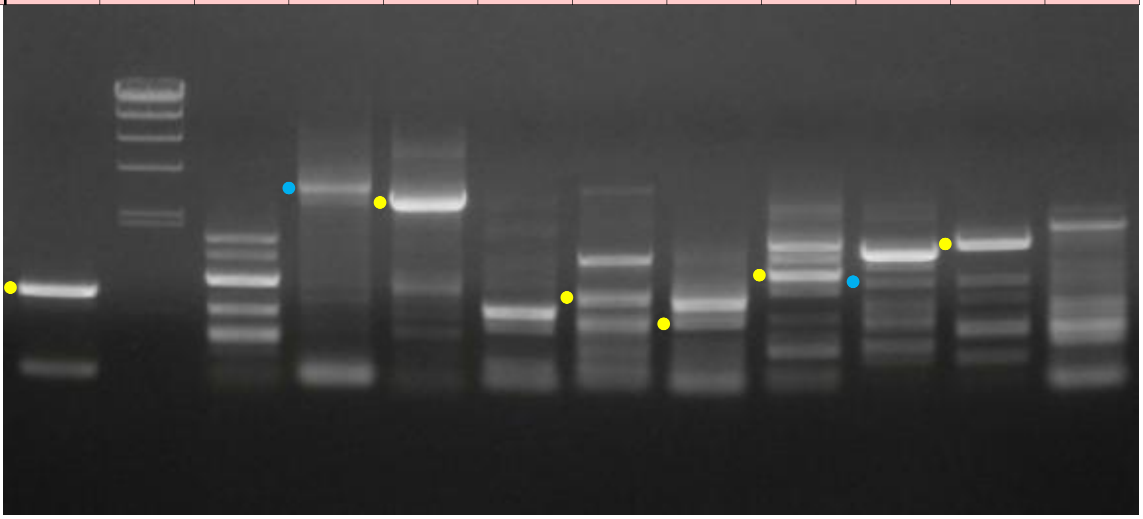

Supplement: S1 Fig — Sample images of PCR products for mKO test cloning and RT-PCR products for cDNA cloning (0.7% agarose gel electrophoresis). (PDF) [file pone.0139349.s001.pdf]
