# Supplementary material for: An Alternative Method to Facilitate cDNA Cloning for Expression Studies in Mammalian Cells by Introducing Positive Blue White Selection in Vaccinia Topoisomerase I-Mediated Recombination
Source: PLoS One. 2015 Sep 30;10(9):e0139349. doi: 10.1371/journal.pone.0139349 (PMC4589362; doi:10.1371/journal.pone.0139349)
Supplement: S2 File — Protocols for cDNA cloning and vector preparation (PDF) [file pone.0139349.s003.pdf]

Supporting Information (File S1)

Preparation of *Vaccinia* topoisomerase I-linked vectors

1. Digest 5 μg of the vector DNA with Nt.*Bsp*QI for 1 hr at 50°C

H_2O	38.5
10x NEBuffer 3	5
100x BSA	0.5
Vector (1 μg/μl)	5
Nt.*Bsp*QI (10 U/μl)	1
	50 μl

* Buffer, BSA, and Nt.*Bsp*QI are from New England Biolab

* Use purified DNA (no inclusion of denatured plasmids which are resistant to digestion)

2. Heat-denature at 80°C for 20 min
3. Spin briefly to pull down moisture
4. Digest with *Eco*RV for 1 hr at 37°C

Reaction mix	50
*Eco*RV (15U/μl)	1
	51 μl

* Digestion may be confirmed by electrophoresis. Uncut vectors increase the number of white colonies on X-gal plates.

5. Use 10 μl for the following reactions

*Store the rest at -20°C for future use

6. React with *Vaccinia* topoisomerase I for 5 min at 37°C

Reaction mix	10
0.5 M EDTA	0.4
Topo（0.2 μg/μl）	1
	11.4 μl

* In this prep, the enzyme used was stoichiometrically 20 times more than the vector DNA (6 pmole of the 32-kDa enzyme and 0.3 pmole of the 5-kb vector DNA)

* Note: I previously used a commercially available enzyme (Epicentre, 10 unit/μl) but it did not work, most likely due to the small amount of enzyme.

7. Put on ice
8. Add PEG

Reaction mix	11
H_2O	9
30 mM $MgCl_2$	20
30 % PEG*	20
	60 μl

* PEG (polyethylene glycol, average molecular weight of 8,000)

9. Centrifuge at 14,000 rpm for 20 min at 4°C
10. Completely remove supernatant and dissolve DNA in 10 μl of 50 mM Tris-HCl (pH 7.5), 100 mM NaCl, 2.5 mM EDTA

    * Dissolve DNA promptly after the removal of supernatant (drying may denature the enzyme)

    * Vector DNA concentration is about 20-40 ng/μl.
11. Use the enzyme-linked vector for cloning
12. Store the rest at -20°

    * It is recommended to check DNA samples by agarose gel electrophoresis (as shown in Figure 4B).

    *The enzyme-linked vector DNAs show electrophoretic mobility shift.

Preparation of PCR-amplified cDNAs (Outlines)

1. Design primers according to the Invitrogen's protocol. Forward primers are about 25 nucleotides in length, including four extra bases (CACC) at the 5'-end of the initiation codon (ATG). Reverse primers are about 21 nucleotides in length, corresponding to the 3'-end sequences of the open reading frame excluding its stop codon.
2. Amplify cDNAs by PCR. Use a proofreading thermostable DNA polymerase. PCR-amplified products must have blunt ends.

    * DNA fragments amplified with *Taq* DNA polymerase is not compatible to this system
3. Gel-purify PCR products of expected size.

    * For large-scale cloning, the method described by Heyman et al (Genome Res, 9:383-, 1999) may be convenient.
4. Check DNA fragments by agarose gel electrophoresis.

    * Make sure DNA concentration and size

***Vaccinia* topoisomerase I-mediated cloning**

1. Prepare the reaction mixture on ice

	Control	+ Insert
H_2O	1.5	1
5x Topo buffer*	0.5	0.5
Vector (20-40 ng/μl)	0.5	0.5
cDNA (10-20 ng/μl)		0.5
	2.5 μl	2.5 μl

    * 5x Topo buffer (500 mM Tris [pH 7.5], 500 mM NaCl, 12.5 mM $MgCl_2$)

    * A higher molar ratio of insert cDNAs reduces the cloning efficiency.
2. React for 10 min at room temperature
3. Put on ice

Transformation

1. Prepare reaction on ice

Competent cell	50
DNA	2.5
	52.5 μl

   * *E.coli* strains harboring *lacZΔM15* (e.g. DH5α) are used for competent cells
2. Incubate for 30 min on ice
3. Heat-shock at 42°C for 1 min
4. Put on ice for 2 min
5. Add 500 μl of 2xYT
   * SOC or LB medium may be used
6. Incubate for 2-3 hr at 37°C
   * For ampicillin resistant vectors, incubate for 1 hr
7. Spread 250 μl on a X-gal plate
   * X-gal plate: LB plate with 30 mg/l kanamycin or 100 mg/l ampicillin, and 40 mg/l X-gal.
   * No addition of isopropyl-β-D-thiogalactopyranoside (IPTG)
8. Incubate for 18-22 hr at 37°C
   * Don't incubate more than 22 hr since negative colonies become bluish due to nonsense suppression.
9. Pick blue colonies for analysis
   * Avoid taking light-blue colonies
   * On average, 90 colonies were obtained (competent cells at ~1 x 10^6 cfu/μg DNA/100 μl cells)
   * Cloning with ampicillin resistant vectors yields 4-8-fold more colonies on X-gal plates
